# ﻿Two new species of the family Aoridae (Crustacea, Malacostraca, Amphipoda) from Korean waters

**DOI:** 10.3897/zookeys.1210.124190

**Published:** 2024-08-20

**Authors:** June Kim, Jae-Hong Choi, Yu-Jin Kim, Hyeong-Woo Im, Young-Hyo Kim

**Affiliations:** 1 Department of Biological Sciences, Dankook University, Cheonan 31116, Republic of Korea Dankook University Cheonan Republic of Korea

**Keywords:** Amphipod, Aoridae, *Aoroides*, *Grandidierella*, Korea, new species

## Abstract

Two new species of the family Aoridae, one from the genus *Aoroides* Walker, 1898, and other from the genus *Grandidierella* Coutière, 1904, are reported from Korean waters. *Aoroidesgracilicrus***sp. nov.** is similar to *A.longimerus* in having numerous plumose setae on the basis and carpus of gnathopod 1. However, the new species can be distinguished from *A.longimerus* by the numerous plumose setae on the bases of pereopods 3 and 4 and the slender basis of pereopod 7. *Grandidierellanaroensis***sp. nov.** is morphologically most similar to *G.pseudosakaensis*. However, the new species can be distinguished by the presence of small distal and proximal processes and a large middle process on the carpus of gnathopod 1, and the subovate propodus of gnathopod 1. Both new species are illustrated and compared to related species. A key to species in the family Aoridae from Korean waters is also provided.

## ﻿Introduction

The family Aoridae Stebbing, 1899 includes amphipod species that are abundant in coastal and sublittoral waters. Aorid amphipods usually feed on generalized organic detritus and algal debris, but some aorids can be opportunistic predators. *Microdeutopusgryllotalpa* Costa, 1853 will seize and consume small crustaceans passing the opening of its tube, and an undescribed species of *Grandidierella* from northwestern Australia has been observed to feed on insect larvae (Myers and Lowry 2003).

The family Aoridae was first established by Stebbing (1899) with *Aoratypica* Krøyer, 1845 as its type species. Aorid amphipods are characterized by an enlarged male gnathopod 1 and are easily distinguished from congeners by pereopod 7, which is disproportionately longer than pereopod 6. According to the ratio of the length of pereopod 7, groups with pereopod 7 relatively longer than pereopods 5 and 6 have been classified as the family Aoridae, whereas groups with pereopods 5–7 of similar length have been classified as the family Unciolidae (Myers and Lowry 2003). In addition, due to strong sexual dimorphism in the family, the female gnathopod 1 is subequal in size to gnathopod 2, without an especially enlarged or elongated article.

The Aoridae includes 244 species in 26 genera (Horton et al. 2024). The genus *Aoroides* Walker, 1898 consists of 20 species and was established by Walker (1898), with *A.columbiae* Walker, 1898 as its type species. *Aoroides* is characterized by the absence of an accessory flagellum and an elongated merus of the male gnathopod 1 (Barnard 1962). The genus *Grandidierella* Coutière, 1904 comprises 52 species. This genus was established by Coutière (1904), with *G.mahafalensis* Coutière, 1904 as its type species. *Grandidierella* is characterized by the enlarged male gnathopod 1 carpus, which typically has 1–3 processes.

In this paper, we add a new species each of the genera *Aoroides* and *Grandidierella* to the Korean aorid amphipod fauna. A total of 32 species belonging to seven genera have been reported to date in Japanese waters adjacent to Korea, including two species of *Aora* Krøyer, 1845, 11 species of *Aoroides*, one species of *Bemlos* Shoemaker, 1925, 13 species of *Grandidierella*, three species of *Paragrandidierella* Ariyama, 2002, one species of *Pseudobemlos* Ariyama, 2004, and one species of *Tethylembos* Myers, 1988 (Ariyama and Kawabe 2022; Ariyama and Kohtsuka 2022). In Chinese waters, 23 species belonging to seven genera have been reported, including one species of *Aora* Krøyer, 1845, two species of *Aoroides*, five species of *Bemlos*, five species of *Globosolembos* Myers, 1985, seven species of *Grandidierella*, two species of *Lembos* Bate, 1856, and one species of *Xenocheira* Haswell, 1879 (Hou and Li 2002; Ren 2006). However, only three species of *Aoroides* and two species of *Grandidierella* have been recorded in Korean waters: *Aoroidescolumbiae* Walker, 1898, *A.ellipticus* Ariyama, 2004, *A.semicurvatus* Ariyama, 2004, *Grandidierellafasciata* Ariyama, 1996, and *G.japonica* Stephensen, 1938 (Kim and Kim 1987; Kim 2011; Jung and Yoon 2013; Jung, Kim and Yoon 2016; Kim and Heo 2016). Therefore, additional species of the family Aoridae are to be expected in Korean waters. This study also provides a key to species in the family Aoridae from Korean waters.

## ﻿Materials and methods

**Figure 1.**
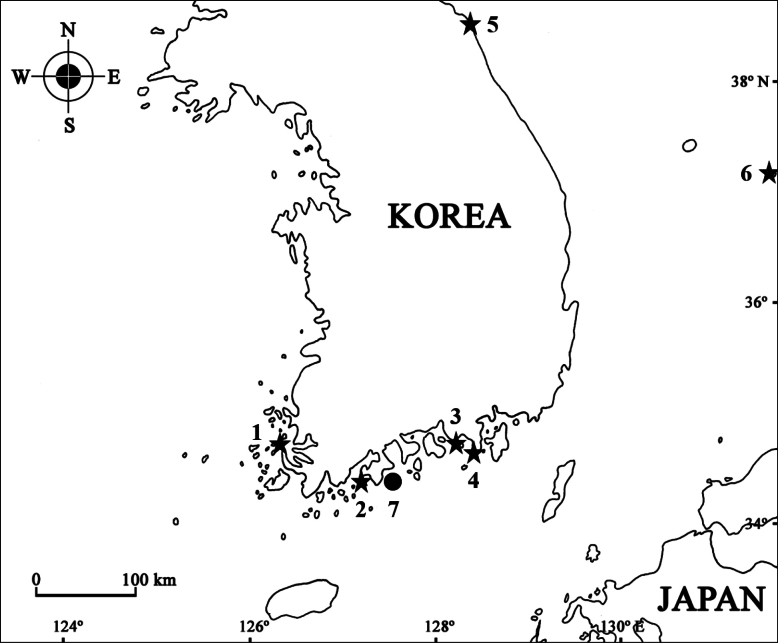
Distribution of the Aoridae species (black star: *Aoroidesgracilicrus* sp. nov., black circle: *Grandidierellanaroensis* sp. nov.; 1, Sogagido Is.; 2, Dolsando Is.; 3, Beol-po port; 4, Bijin-ri; 5, Daejin port; 6, Dokdo Island; 7, Narodo Island).

Specimens were collected with a light trap and hand net from subtidal waters of the East and South Sea, Korea (Fig. 1). The specimens were fixed in 95% ethanol and dissected in glycerol on Cobb’s aluminum hole slides. The materials were examined under stereoscopic (Olympus SZX 10) and compound microscopes (Olympus BX 51), and drawings and measurements were made with the aid of a drawing tube. Body length was measured from the end of the rostrum to the end of the urosome, along the dorsal parabolic line of the body. The examined specimens are deposited at the Honam National Institute of Biological Resources (**HNIBR**), Mokpo, Korea, and the Department of Biological Science, Dankook University (**DKU**), Cheonan, Korea.

## ﻿Taxonomy

**Order Amphipoda Latreille, 1816**

**Family Aoridae Stebbing, 1899**

### ﻿Genus Aoroides

Taxon classificationAnimaliaAmphipodaAoridae

Walker, 1898

70DF1C7B-FE66-5EE2-95E9-E09EFF9635D7

#### Type species.

*Aoroidescolumbiae* Walker, 1898.

### ﻿ Aoroides gracilicrus sp. nov.

Taxon classificationAnimaliaAmphipodaAoridae

68AC95EF-1443-59DD-A2AF-E420FBAF3671

https://zoobank.org/DACEFB72-5A24-4CD4-A7CF-3E9CFEAD88A0

**Figure 2.**
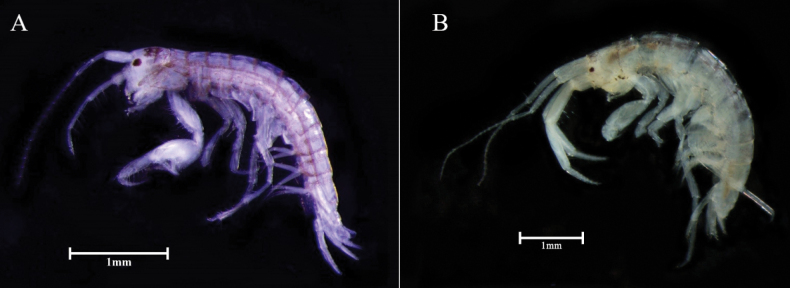
*Aoroidesgracilicrus* sp. nov. **A** male, 3.3 mm **B***Grandidierellanaroensis* sp. nov., male, 4.9 mm. Scale bars: 1.0 mm (**A, B**).

**Figure 3.**
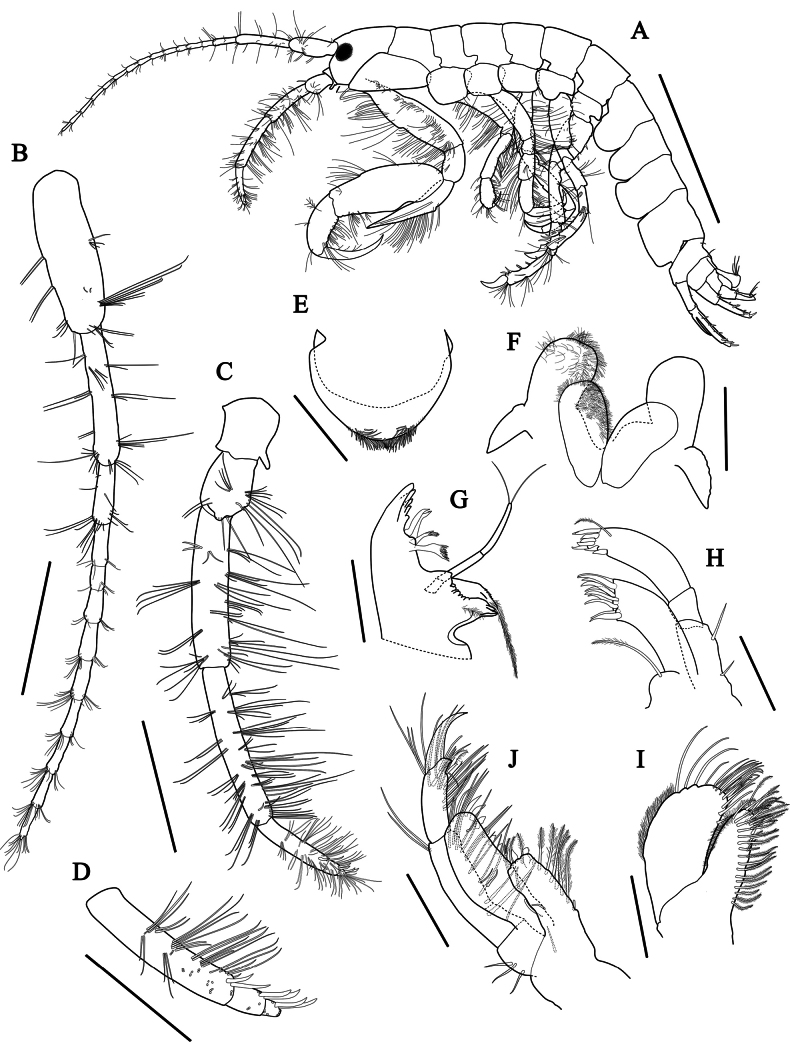
*Aoroidesgracilicrus* sp. nov., holotype, male, 3.3 mm **A** habitus, lateral view **B** antenna 1 **C** antenna 2 **D** flagellum of antenna 2 **E** upper lip **F** lower lip **G** mandible **H** maxilla 1 **I** maxilliped **J** maxilla 2. Scale bars: 1.0 mm (**A**); 0.3 mm (**B–D**); 0.1 mm (**E–J**).

**Figure 4.**
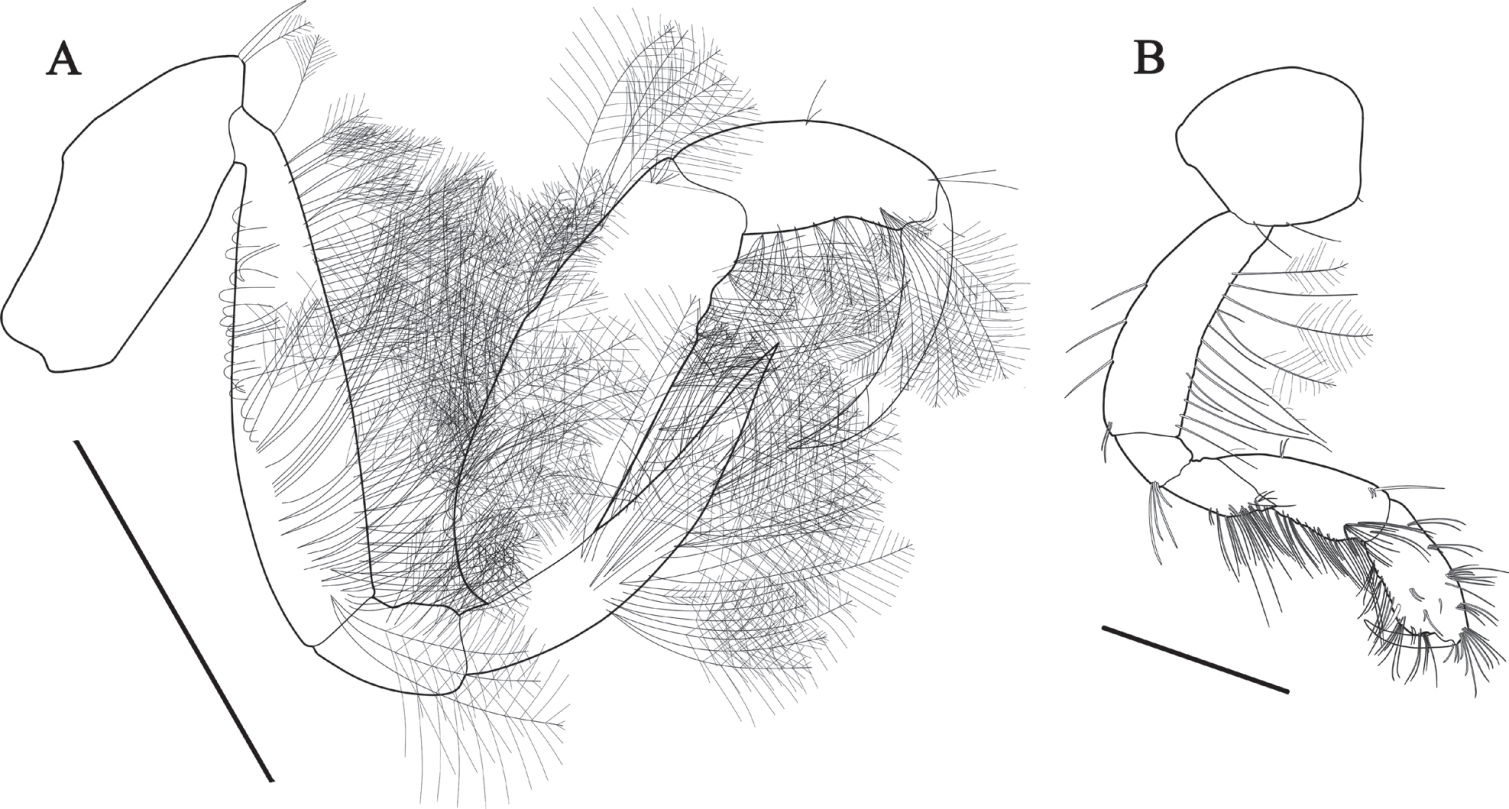
*Aoroidesgracilicrus* sp. nov., holotype, male, 3.3 mm **A** gnathopod 1 **B** gnathopod 2. Scale bars: 0.6 mm (**A**); 0.3 mm (**B**).

**Figure 5.**
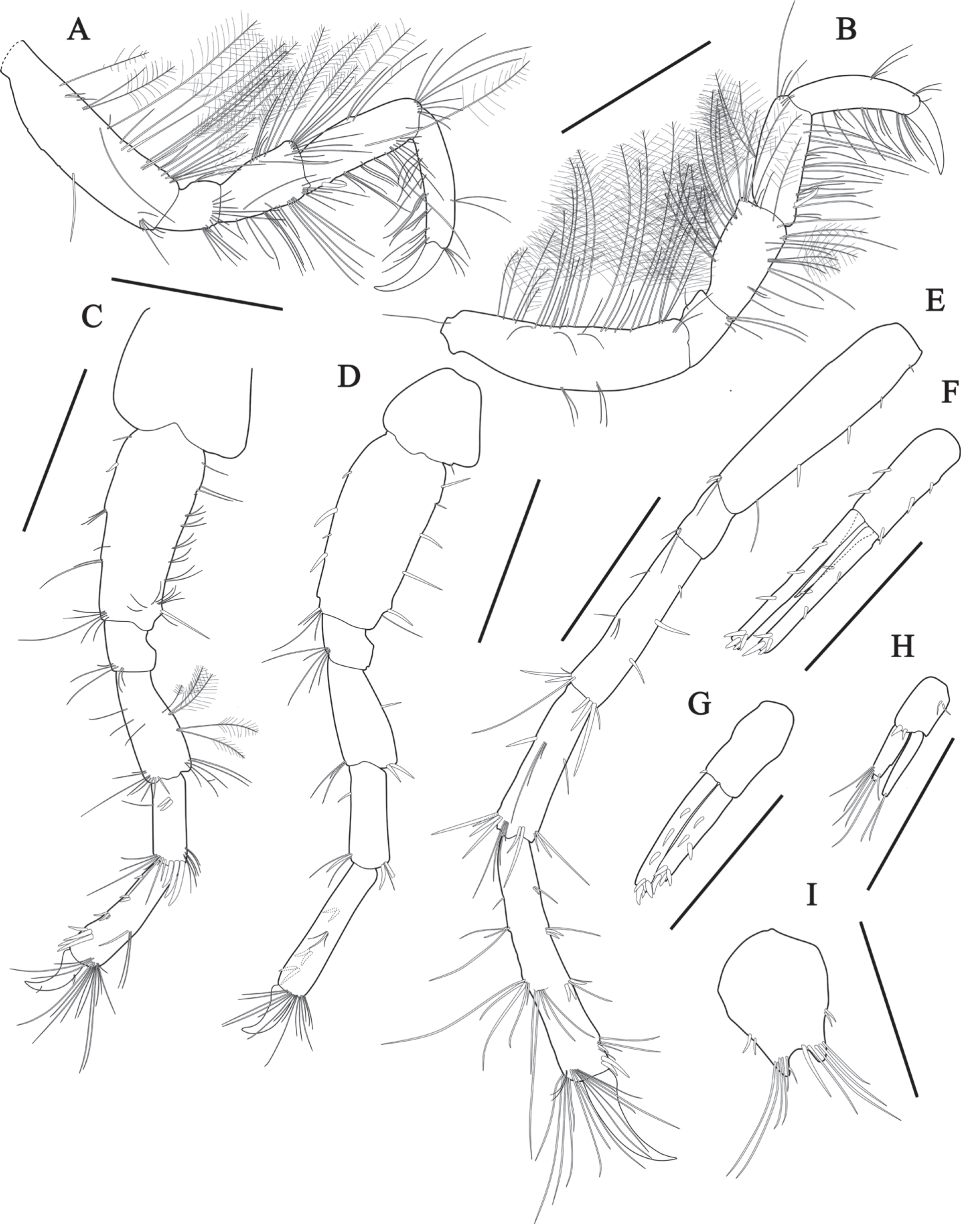
*Aoroidesgracilicrus* sp. nov., holotype, male, 3.3 mm **A** pereopod 3 **B** pereopod 4 **C** pereopod 5 **D** pereopod 6 **E** pereopod 7 **F** uropod 1 **G** uropod 2 **H** uropod 3 **I** telson. Scale bars: 0.3 mm (**A–H**); 0.2 mm (**I**).

Figs 2A , 3 , 4 , 5 Korean name: Ga-neun-da-ri-teol-keun-ap-son-yeop-sae-u, new

#### Type material.

***Holotype***, South Korea • 3.3 mm • 1 ♂; Bijin-ri, Han-san-myeon, Tongyeong-si, Gyeongsangnam-do; 34°42'59"N, 128°27'37"E; 1 August 2019; collected from hand net; Y.H. Kim leg.; HNIBRIV2426.

#### Additional material.

South Korea • 3.1 mm • 1 ♂; Dolsando Island, Yeosu-si, Goheung-gun, Jeollanam-do; 34°37'19"N, 127°47'55"E; 20 March 2004; collected from hand net; Y.H. Kim leg., DKUAMP202402 • 1 ♂; Beol-po port, Sanyang-eup, Tongyeong-si, Gyeongsangnam-do; 34°49'56"N, 128°21'21"E, collected from light trap; 24 August 2005; Y.H. Kim • 1 ♂; Sogagido Island, Nagwol-myeon, Yeonggwang-gun, Jeollanam-do; 35°15'08"N, 126°06'04"E; collected from hand net; 27 June 2007; Y.H. Kim leg. • 1 ♂; Daejin port, Hyeonnae-myeon, Goseong-gun, Gangwon-do; 38°29'56"N, 128°25'34"E; collected from light trap; 11 June 2009; Y.H. Kim • 1 ♂; Dokdo Island, Ulleung-eup, Ulleung-gun, Gyeongsangbuk-do; 37°26'01"N, 131°56'52"E; collected from hand net; 22 February 2019; Y.H. Kim leg.

#### Diagnosis.

Eyes well developed, oval. Antenna 1 slender, except peduncular article 1, elongated, moderately setose; flagellum longer than peduncle. Antenna 2 densely setose, stout, gland cone bluntly pointed; flagellum short, 3-articulate, with two robust setae on each article distally. Mandible, incisor with five dentate, tricuspidate lacinia mobilis, molar triturative. Maxilla 1, outer plate with 10 dentate setae apically. Gnathopod 1 merochelate, massive, greatly larger than gnathopod 2, covered by plumose setae; coxa with a long robust and a plumose seta on anteroventral corner. Pereopod 7 slender, elongated, longer than pereopods 5 and 6. Uropod 1 with a large inter-ramal process. Uropod 3, outer ramus biarticulate. Telson short, fleshy, truncate distally.

#### Description.

**Holotype, adult male**, HNIBRIV2426.

***Body*** (Figs 2A, 3A) 3.3 mm long, dorsally smooth, laterally compressed; eyes well developed, oval.

***Antenna 1*** (Fig. 3B) slender, elongated, moderately setose, length ratio of peduncular articles 1–3 = 1.00: 0.95: 0.43; flagellum 10-articulate, terminal article minute, length 0.87 × peduncle; accessory flagellum absent.

***Antenna 2*** (Fig. 3C) densely setose; gland cone bluntly pointed, short; peduncular articles 1–3 short, peduncular articles 4, 5 subrectangular; length ratio of peduncular articles 3–5 = 1.00: 1.22: 0.41; flagellum (Fig. 3D) 3-articulate, with two robust setae distally on each article.

***Upper lip*** (Fig. 3E) subovate, apical margin round and pubescent.

***Lower lip*** (Fig. 3F), inner plate elongate-ovate, densely pubescent medially and distally; outer plate distally expanded, mediodistal margin round and pubescent; mandibular process developed.

***Left mandible*** (Fig. 3G), incisor with 5 blunt teeth and lacinia mobilis with 3–4 teeth; accessory setal row composed of two multicuspidate setae; molar triturative with pappose seta; palp slender, 3-articulate; article 1 short, unarmed, length 0.28 × article 3; article 2 unarmed, length 0.84 × article 3; article 3 with two long setae distally.

***Maxilla 1*** (Fig. 3H), inner plate small with long pinnate seta apically; outer plate, apical margin with 10 dentate setal teeth; palp biarticulate, article 1 short, unarmed, article 2 swollen distally, apex round and weakly serrate, with seven robust setae, subapical portion with one pinnate seta.

***Maxilla 2*** (Fig. 3I), inner plate slender, slightly narrowing distally, apex and medial margin setose, with oblique row of plumose setae on surface; outer plate large, broad, with row of mediodistal setae, apex round, slightly notched subapically, pubescent laterally.

***Maxilliped*** (Fig. 3J), inner plate subrectangular, medial margin with seven plumose setae submarginally, apex with four plumose and two stout robust setae; outer plate subrectangular, apex expending beyond end of palp article 2, medial margin straight with seven blunt robust setae gradually increasing in size distally and two elongate setae apically; palp 4-articulate, article 1 short, articles 2 and 3 subrectangular, medial and laterodistal margins lined with long setae, article 4 falcate, with a nail.

***Gnathopod 1*** (Fig. 4A) merochelate, densely setose, moderate, greatly larger than gnathopod 2; coxa wider than long, width 2.51× length, with one robust and one plumose setae on anteroventral corner; basis relatively long, slender, moderately broad, anterior and lateral margins with numerous plumose setae, posterior margin with 17 setae; ischium short, length 0.28× basis, with five plumose setae anterolaterally; merus elongated, located below carpus, length 0.86× basis, apex acute, ventral surface with numerous plumose setae; carpus moderate, subrectangular, length 1.05× merus, anterior and posterior margins with numerous plumose setae; propodus slightly curved anteriorly, length 0.57× carpus, posterior margin with numerous plumose setae; dactylus elongated, falcate, length 1.14× propodus.

***Gnathopod 2*** (Fig. 4B) subchelate; coxa subquadrate, width 1.11× length; basis subrectangular, width 0.32 × length, slightly curved posteriorly, anterior margin with plumose and simple setae, posterior margin with simple setae; merus 1.25× ischium, half of posterodistal margin setose; carpus subrectangular, widening distally, ventrally setose; propodus subrectangular, length 0.83× carpus, anterior margin with five clusters of setae and posterior margin setose, palm steeply angled, defined by one robust seta; dactylus falcate, overreaching palm, length 0.66× propodus.

***Pereopod 3*** (Fig. 5A) setose; basis subrectangular, width 0.27× length, anterior margin with 13 plumose and 10 simple setae, posterior margin with six simple setae; merus subquadrate, length 1.82× ischium, with seven plumose and 12 simple setae anteriorly, 19 simple setae posteriorly; carpus subrectangular, length 1.29× merus, with one robust seta posteroproximally; propodus rectangular, slender, length 1.04× carpus, posterior margin setose; dactylus falcate, length 0.49× propodus.

***Pereopod 4*** (Fig. 5B) similar to pereopod 3, but merus with plumose setae along with simple setae on posterior margin.

***Pereopod 5*** (Fig. 5C), coxa bilobate, wider than long; basis subrectangular, slightly narrowing distally, length 2.52× width, both margins with unequal simple setae; merus widening distally, length 1.59× ischium, posterior margin with five plumose and four simple setae; carpus rectangular, length 0.77× merus, with two proximal and three distal robust setae; propodus slender, slightly widening distally, length 1.52× carpus, anterior margin with 1-2-3-4 robust setae formula; dactylus falcate, length 0.48× propodus.

***Pereopod 6*** (Fig. 5D) similar to pereopod 5, but less setose, unarmed.

***Pereopod 7*** (Fig. 5E) elongated, much longer than either pereopods 5 or 6; basis characteristic in form, subrectangular, slender, width 0.14× length; anterior margin with four robust and one simple setae; ischium short, length 0.23× basis, posterodistal corner with two setae; merus rectangular, length 2.29× ischium; carpus rectangular, length 0.86× merus; propodus elongate, length 1.64× carpus; dactylus falcate, length 0.43× propodus.

***Uropod 1*** (Fig. 5F), peduncle subrectangular, length 0.73× inner ramus, with three dorsolateral and two dorsomedial robust setae, and a large inter-ramal process; outer ramus length× 1.09 inner ramus, with two robust setae on both margins, four robust setae on apex; inner ramus with three dorsal and four apical robust setae.

***Uropod 2*** (Fig. 5G), peduncle subrectangular, subequal in length to inner ramus, with one dorsomedial robust seta; outer ramus length 1.26× inner ramus, with three dorsal and four apical robust setae; inner ramus with three dorsal and four apical robust setae.

***Uropod 3*** (Fig. 5H) short, length 0.52× uropod 2; peduncle subequal to outer ramus, with two dorsolateral and two dorsomedial robust setae; outer ramus 0.73× inner ramus, biarticulate, proximal article with six long setae subapically, terminal article minute, with two long setae on apex; inner ramus with two long setae on apex.

***Telson*** (Fig. 5I) short and fleshy, longer than broad, concave distally, each lobe with two lateral robust setae and 5–6 unequal setae near the apex.

**Female.** Unknown.

#### Remarks.

The new species *Aoroidesgracilicrus* sp. nov. resembles *A.longimerus* Ren & Zheng, 1996 from Dayawan, China, *A.myojinensis* Ariyama, 2004 from Myojin-zaki, Japan, and *A.secunda* Gurjanova, 1951 from Primorskii Krai, Russia, in having densely setose anterior margins of the basis and carpus of gnathopod 1. However, *A.gracilicrus* sp. nov. is distinguished from its congeners by the characters and character states shown in Table 1 and the following features (different characters and character states of *A.longimerus*, *A.myojinensis*, and *A.secunda* in brackets): 1) gnathopod 1, coxa with an anterior robust and a plumose setae (vs four plumose setae and a robust seta in *A.longimerus*, unarmed in *A.myojinensis*, and two robust setae in *A.secunda*); 2) gnathopod 1, carpus slender and subrectangular (vs moderate in *A.longimerus*, and ovate in *A.myojinensis* and *A.secunda*); 3) pereopods 3 and 4, anterior margins setose with plumose setae (vs sparse with simple setae in *A.longimerus* and *A.myojinensis*); 4) pereopod 3, carpus with a robust seta posteriorly (vs two robust setae in *A.longimerus*, five robust setae in *A.myojinensis*, and three robust setae in *A.secunda*); 5) pereopod 7, basis slender and subrectangular (vs elongate ovate in *A.longimerus*, *A.myojinensis*, and *A.secunda*); and 6) uropod 3, both rami unarmed (vs both rami with robust setae in *A.longimerus* and *A.myojinensis*).

**Table 1.** Morphological characters of *Aoroidesgracilicrus* sp. nov. and related species with numerous plumose setae on anterior margin of basis and carpus of gnathopod 1.

Species Characters	*A.longimerus*	*A.myojinensis*	*A.secunda*	*A.gracilicrus* sp. nov.
Body length	3.6 mm	2.8 mm	3.9 mm	3.3 mm
Coxa 1	1 robust seta & 4 plumose setae	Unarmed	2 long robust setae	1 robust seta & 1 plumose seta
Gnathopod 1, carpus	Moderate	Ovate	Elongate ovate	Subrectangular
Pereopod 3, anterior margin of basis	Sparse simple setae	Sparse simple setae	Dense Plumose setae	Dense Plumose setae
Pereopod 3, posterior margin of carpus	2 robust setae	5 robust setae	3 robust setae	1 robust seta
Pereopod 4, anterior margin of basis	Sparse simple setae	Sparse simple setae	Dense Plumose setae	Dense Plumose setae
Pereopod 7, basis	Elongate ovate	Elongate ovate	Elongate ovate	Slender, Subrectangular
Uropod 3, both rami	Lateral robust setae	Lateral robust setae	Unarmed	Unarmed
Distribution	Osaka, Japan	Myojin-zaki, Japan	Primorskii Krai, Russia	Bijin-ri, Korea
Reference	Ariyama 2004	Ariyama 2004	Gurjanova 1951	Present study

#### Etymology.

The species name is derived from the Latin *gracilis* (= slender) and *crus* (= leg) in reference to the relatively slender basis of pereopod 7.

#### Distribution.

Korea (East Sea, South Sea).

### ﻿Genus Grandidierella

Taxon classificationAnimaliaAmphipodaCorophiidae

Krøyer, 1845

2BB91A4C-A000-5311-9638-92F0255BE22C

#### Type species.

*Grandidierellamahafalensis* Coutière, 1904.

### ﻿ Grandidierella naroensis sp. nov.

Taxon classificationAnimaliaAmphipodaCorophiidae

6F74C8AE-3056-55A8-A7B1-050D9E5BB502

https://zoobank.org/436FEB52-FEF7-447A-9E3B-F2EE9C532141

**Figure 6.**
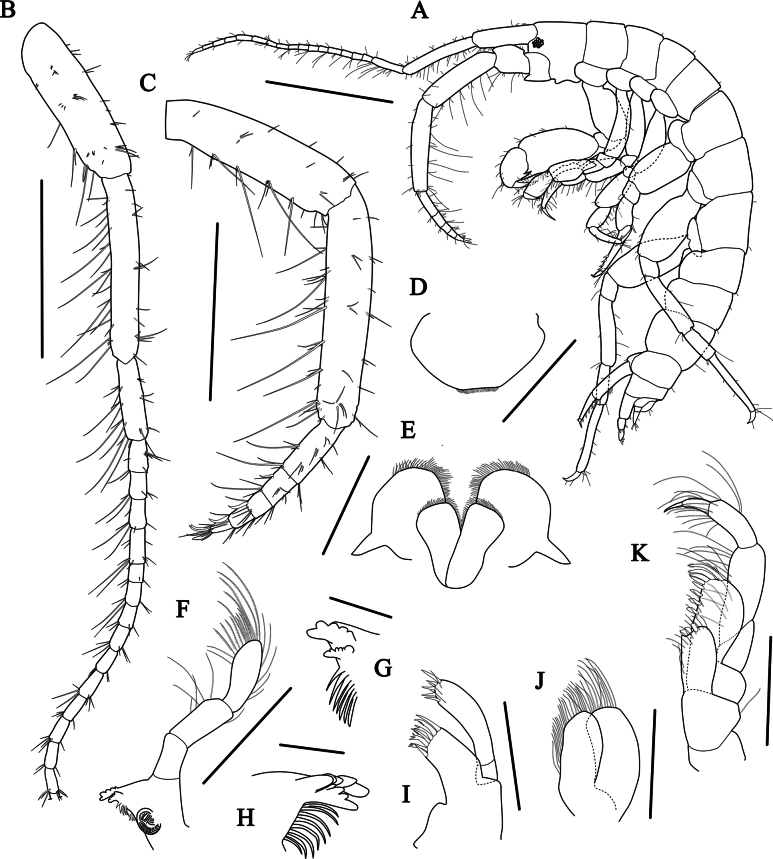
*Grandidierellanaroensis* sp. nov., holotype, male, 4.9 mm **A** habitus **B** antenna 1 **C** antenna 2 **D** upper lip **E** lower lip **F** right mandible **G** right mandible teeth **H** left mandible teeth **I** maxilla 1 **J** maxilliped **K** maxilla 2. Scale bars: 1.0 mm (**A**); 0.4 mm (**B, C, F**); 0.2 mm (**D, E, I–K**); 0.05 mm (**G, H**).

**Figure 7.**
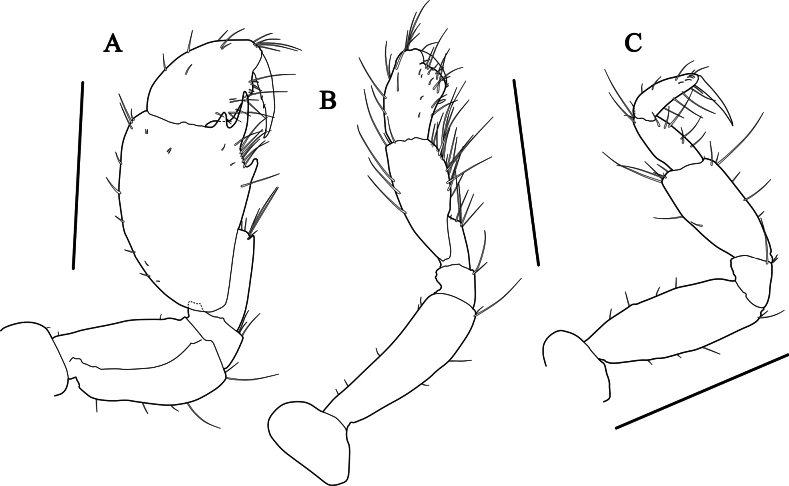
*Grandidierellanaroensis* sp. nov., holotype, male, 4.9 mm **A** gnathopod 1 **B** gnathopod 2 **C** pereopod 3. Scale bars: 0.4 mm (**A–C**).

**Figure 8.**
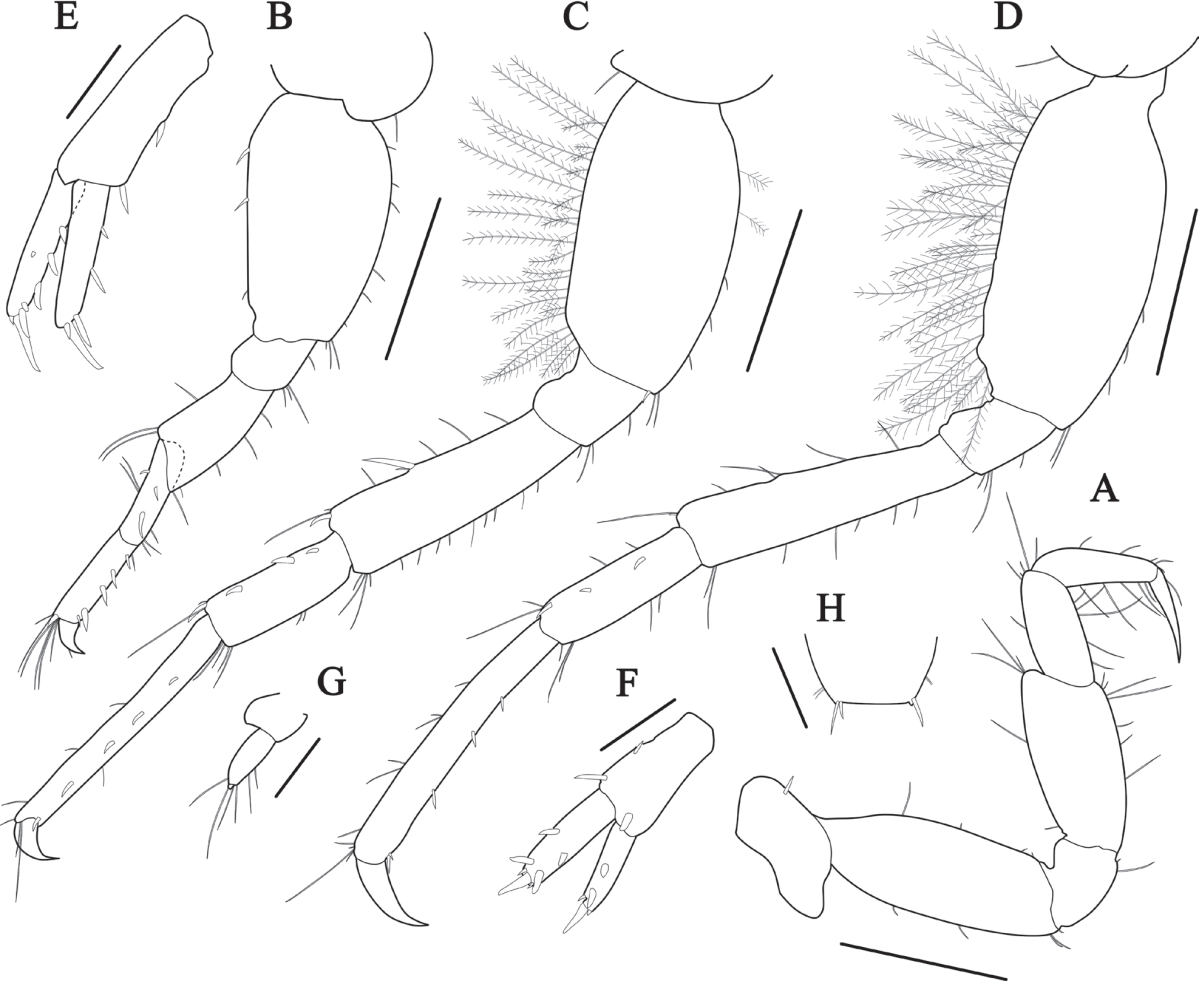
*Grandidierellanaroensis* sp. nov., holotype, male, 4.9 mm **A** pereopod 4 **B** pereopod 5 **C** pereopod 6 **D** pereopod 7 **E** uropod 1 **F** uropod 2 **G** uropod 3 **H** telson. Scale bars: 0.2 mm (**A–D**); 0.1 mm (**E–H**).

Figs 2B , 6 , 7 , 8 Korean name: Na-ro- do-keun-ap-son-yeop-sae-u, new

#### Type material.

***Holotype***, South Korea • 4.9 mm • 1 ♂; Yeom-po beach, Narodo Island, Goheung-gun, Jeollanam-do; 34°25'57"N, 127°29'31"E; collected from hand net; 01 April 2022; Y.H. Kim leg.; HNIBRIV2427.

#### Diagnosis.

Antenna 1 slender, elongated. Antenna 2 relatively stout, flagellum short. Gnathopod 1 carpochelate, enlarged, ischium without posterodistal process, merus with weak process posterodistally, carpus with three processes on posterodistal corner, distal and proximal processes short, middle process elongate. Gnathopod 2, carpus longer than propodus, palm steeply angled. Pereopod 6, basis anterior margin with two plumose and three setae. Uropod 1, peduncle lacking inter-ramal process ventrodistally. Uropod 3 uniramus, ramus biarticulate.

#### Description.

**Adult male**, HNIBRIV2427.

***Body*** (Figs 2B, 6A) 4.9 mm long, subcylindrical, dorsally smooth; eye small, rounded, composed of ommatidia.

***Antenna 1*** (Fig. 6B) slender, elongated; length ratio of peduncular articles 1–3 = 1.00: 1.03: 0.44; flagellum 16-articulate; accessory flagellum short, 2-articulate.

***Antenna 2*** (Fig. 6C) shorter than antenna 1; peduncular articles 4 and 5 subrectangular, setose; length ratio of peduncular articles 3–5 = 1.00: 2.85: 3.14; flagellum short, 5-articulate, each article with 1–2 robust setae ventrally.

***Upper lip*** (Fig. 6D) subrounded, apical margin truncate and pubescent.

***Lower lip*** (Fig. 6E), inner plate elongate-ovate, pubescent medially and distally; outer plate expanded, distal margin rounded and pubescent; mandibular process well developed.

***Right mandible*** (Figs 6F, G), incisor with five blunt teeth and lacinia mobilis with six teeth; accessory setal row composed of seven setae; molar triturative; palp stout, 3-articulate; article 1 relatively short, unarmed, length 0.58 × article 3; article 2 with five setae medially and one seta laterally, length 0.77× article 3; article 3 with 13 unipectinate and two simple setae distally and five simple setae laterally.

***Left mandible*** (Fig. 6H) similar to right mandible, but lacinia mobilis with four teeth and accessory setal row composed of eight setae.

***Maxilla 1*** (Fig. 6I), inner plate vestigial; outer plate, apical margin with eight dentate setal teeth (two simple, one bifid, two tricuspidate, and three multicuspidate); palp biarticulate, article 1 short, unarmed, article 2 swollen distally, apex round, with five robust and three simple setae.

***Maxilla 2*** (Fig. 6J), inner plate apex and medial margins setose; outer plate subequal to inner plate, with row of distal setae.

***Maxilliped*** (Fig. 6K), inner plate subrectangular, mediodistal corner slightly produced, medial margin with five setae, apex with six simple and three stout robust setae; outer plate elongate-ovate, medial margin straight with four blunt robust and four simple setae, apical margin with four robust setae gradually increasing in size; palp 4-articulate, article 1 subtriangular, article 2 subrectangular, medial margin with long setae, article 3 slender, lateral margin with two setae, subapical margin with 10 setae, article 4 falcate, with a nail.

***Gnathopod 1*** (Fig. 7A) carpochelate, enlarged; coxa small, subquadrate, width 1.36× length; basis subrectangular, length 1.67× width, expended posteriorly, widening distally; ischium short, length 0.12× basis, without posterodistal process; merus located below carpus, with weak process posterodistally; carpus characteristic in form, massive, length 1.54× width, length 2.01× merus, with three processes on posterodistal corner, distal and proximal processes short, middle process elongate, with seven setae between middle and proximal processes; propodus subovate, width 0.67× length, convex posteriorly, length 0.65× carpus; dactylus falcate, posterior margin with two accessory teeth.

***Gnathopod 2*** (Fig. 7B), coxa subquadrate; basis slender, subrectangular, widening distally; merus located below carpus, length 1.42× ischium; carpus moderate, elongate-ovate, posterior margin setose; propodus subquadrate, length 0.74× carpus, posterior margin with three robust setae, palm steeply angled, defined by two robust setae; dactylus falcate, fitting palm.

***Pereopod 3*** (Fig. 7C), coxa subovate; basis elongate, slightly widened in the middle, both margins with four anterior and three posterior short setae; merus widening distally, length 0.71× basis; carpus slender, short, length 0.59× merus; propodus slender, narrowing distally, subequal to carpus; dactylus falcate.

***Pereopod 4*** (Fig. 8A) similar to pereopod 3, but coxa with one robust seta on ventral margin.

***Pereopod 5*** (Fig. 8B), coxa wider than long, bilobate; basis subovate, length 1.72× width, anterior margin with a row of six short setae, posterior margin with three robust setae; merus subrectangular, widening distally, length 1.88× ischium; carpus slender, length 0.80× merus, with two robust setae; propodus slender, length 1.33× carpus, anterior margin with a row of five robust setae; dactylus short, falcate.

***Pereopod 6*** (Fig. 8C), coxa similar but slightly smaller than coxa 5; basis elongate-ovate, length 1.91× width, anterior margin with two plumose and three setae, posterior margin with row of unequal plumose setae; merus subrectangular, length 0.84× basis, posterior margin with one robust and five simple setae, posterodistal corner with one long and two robust setae; carpus with three robust setae on posterior margin, posterodistal corner with one robust and three setae; propodus slender, length 1.79× carpus, with a row of five robust setae anteriorly; dactylus falcate.

***Pereopod 7*** (Fig. 8D) similar to pereopod 6, but slightly longer, coxa small, weakly bilobate.

***Uropod 1*** (Fig. 8E), peduncle subrectangular, with two dorsolateral robust setae, ventrodistal end lacking inter-ramal process, length 1.27× inner ramus; outer ramus with two dorsal and three terminal robust setae; inner ramus subequal to outer ramus, with three dorsal and four terminal robust setae.

***Uropod 2*** (Fig. 8F), peduncle short, subrectangular, subequal to inner ramus in length, with two dorsolateral and one dorsomedial robust setae; outer ramus with three dorsal and four terminal robust setae; inner ramus subequal to outer ramus, with two dorsal and three terminal robust setae.

***Uropod 3*** (Fig. 8G) uniramus, peduncle short, length 0.83× ramus, unarmed; ramus biarticulate, proximal article with five setae, terminal article minute, with one long seta on apex.

***Telson*** (Fig. 8H) entire, short, broader than long, truncate distally, each lobe with two subapical setae and 1–2 unequal robust setae on the apex.

#### Remarks.

*Grandidierellanaroensis* sp. nov. is morphologically similar to *G.pseudosakaensis* Ariyama, 2020, *G.osakaensis* Ariyama, 1996, and *G.fasciata* Ariyama, 1996 in the following characters and character states: 1) gnathopod 1, merus with a small posterodistal notch; 2) gnathopod 1, carpus with three processes posterodistally. However, *G.naroensis* sp. nov. is distinguished from its congeners by the characters and character states listed in Table 2 and the following features (different characteristics of *G.pseudosakaensis*, *G.osakaensis*, and *G.fasciata* in brackets): 1) maxilla 1, outer plate with eight teeth (vs with nine teeth in *G.pseudosakaensis* and with 10 teeth in *G.fasciata*); 2) gnathopod 1, ischium without a posterodistal process (vs with a process in *G.osakaensis*); 3) gnathopod 1, carpus with small distal and proximal processes and a large middle process (vs with large middle and proximal processes and a small distal process in *G.pseudosakaensis*, *G.osakaensis*, and *G.fasciata*); 4) gnathopod 1, propodus subovate (vs subrectangular in *G.pseudosakaensis*, *G.osakaensis*, and *G.fasciata*); 5) gnathopod 2, posterior margin of propodus with 3 robust setae (vs with 5 robust setae in *G.pseudosakaensis* and *G.osakaensis* and with 4 robust setae in *G.fasciata*); 6) pereopod 7, basis anterior margin without plumose setae (vs with plumose setae in *G.pseudosakaensis*, *G.osakaensis*, and *G.fasciata*), and 7) uropod 1, peduncle without an inter-ramal process (vs with an inter-ramal process in *G.fasciata*).

**Table 2.** Morphological characters of *Grandidierellanaroensis* sp. nov. and related species with three processes on posterodistal margin of carpus of gnathopod 1.

Species Characters	*G.pseudosakaensis*	*G.osakaensis*	*G.fasciata*	*G.naroensis* sp. nov.
Body length	3.9 mm	6.9 mm	8.4 mm	4.9 mm
Maxilla 1, outer plate	9 teeth	8 teeth	10 teeth	8 teeth
Gnathopod 1, ischium posterodistal process	Absent	Present	Absent	Absent
Gnathopod 1, merus posterodistal process	Prominently produced	Prominently produced	Prominently produced	Weakly produced
Gnathopod 1, carpus	Large middle and proximal processes and small distal process	Large middle and proximal processes and small distal process	Large middle and proximal processes and small distal process	Small distal and proximal processes and large middle tooth
Gnathopod 1, propodus	Subrectangular	Subrectangular	Subrectangular	Subovate
Gnathopod 2, propodus posterior margin	5 robust setae	5 robust setae	4 robust setae	3 robust setae
Pereopod 7, basis anterior margin	With plumose setae	With plumose setae	With plumose setae	Without Plumose setae
Uropod 1, peduncle inter-ramal process	Absent	Absent	Present	Absent
Distribution	Iriomote island, japan	Osaka, japan	Osaka, japan	Narodo island, korea
Reference	Ariyama 2020	Ariyama 1996	Ariyama 1996	Present study

#### Etymology.

The species name is derived from the type locality, Narodo Island, located on the south coast of Korea.

#### Distribution.

Korea (Narodo Island).

### ﻿Key to species of the family Aoridae from Korean waters

**Table:** 

1	Antenna 1, accessory flagellum present and elongate	***Aorapseudotypica* Hirayama, 1984**
–	Antenna 1, accessory flagellum absent or short	**2**
2	Gnathopod 1 carpochelate; uropod 3 uniramus	**3**
–	Gnathopod 1 merochelate; uropod 3 biramus	**6**
3	Antenna 1, accessory flagellum absent; maxilliped, inner plate without distal robust seta	***Paragrandidierellaminima* Ariyama, 2002**
–	Antenna 1, accessory flagellum present; maxilliped, inner plate with distal robust seta	**4**
4	Gnathopod 1, carpus with one posterodistal and two medial processes	***Grandidierellajaponica* Stephensen, 1938**
–	Gnathopod 1, carpus with three posteromarginal processes	**5**
5	Gnathopod 1, propodus subrectangular; uropod 1, peduncle with inter-ramal process	***Grandidierellafasciata* Ariyama, 1996**
–	Gnathopod 1, propodus subovate; uropod 1, peduncle without inter-ramal process	***Grandidierellanaroensis* sp. nov.**
6	Gnathopod 1 weakly setose; gnathopod 2, propodus posterior margin curved	***Aoroidessemicurvatus* Ariyama, 2004**
–	Gnathopod 1 densely setose; gnathopod 2, propodus posterior margin straight	**7**
7	Gnathopod 1, basis and carpus with simple setae; pereopod 3, basis without plumose setae	***Aoroidespunctatus* Ariyama, 2004**
–	Gnathopod 1, basis and carpus with plumose setae; pereopod 3, basis with plumose setae	**8**
8	Gnathopod 1, coxa with one robust seta; pereopod 7, basis elliptical	***Aoroidesellipticus* Ariyama, 2004**
–	Gnathopod 1, coxa with one robust and one plumose setae; pereopod 7, basis subrectangular	***Aoroidesgracilicrus* sp. nov.**

## Supplementary Material

XML Treatment for Aoroides

XML Treatment for Aoroides gracilicrus

XML Treatment for Grandidierella

XML Treatment for Grandidierella naroensis

## ﻿Additional information

### Author ORCIDs

June Kim https://orcid.org/0000-0002-4758-4117

Jae-Hong Choi https://orcid.org/0000-0003-4536-8792

Young-Hyo Kim https://orcid.org/0000-0002-7698-7919

### Data availability

All of the data that support the findings of this study are available in the main text.
